# Human and Robot Assistance for Cognitive Load in Younger and Older Adults: Multimodal Within-Subject Experimental Study

**DOI:** 10.2196/94738

**Published:** 2026-06-01

**Authors:** Simone Varrasi, Roberto Vagnetti, Nicola Camp, John Hough, Alessandro Di Nuovo, Sabrina Castellano, Daniele Magistro

**Affiliations:** 1 Department of Educational Sciences, University of Catania, Catania, Sicily, Italy; 2 Kent and Medway Medical School, University of Kent and Canterbury Christ Church University, Canterbury, England, United Kingdom; 3 Department of Sport Science, School of Science and Technology, Nottingham Trent University, Nottingham, England, United Kingdom; 4 Department of Computing, Sheffield Hallam University, Sheffield, England, United Kingdom; 5 School of Health Sciences, University of Southampton, Building 67, Highfield Campus, University Road, Southampton, England, SO17 1BJ, United Kingdom

**Keywords:** aging, assistive robotics, cognitive load, daily autonomy, multimodal measures

## Abstract

**Background:**

Maintaining cognitive efficiency and independence is a central goal of healthy aging. Socially assistive robots (SARs) are increasingly proposed as scalable digital health solutions to support daily activities in older adults and to facilitate aging-in-place. However, concerns remain regarding whether robot-mediated assistance reduces or inadvertently increases cognitive load, potentially undermining usability, user acceptance, and long-term real-world adoption, particularly in aging populations.

**Objective:**

This study aimed to examine how robot-assisted (human-robot interaction [HRI]) and human-assisted (human-human interaction [HHI]) support influences cognitive load during task performance in younger and older adults. A multimodal assessment framework integrating behavioral, subjective, and physiological measures was used to identify age-related differences in cognitive effort and stress associated with different forms of assistance.

**Methods:**

A total of 60 healthy adults (30 younger adults: mean age 34.8, SD 10.1 years; and 30 older adults: mean age 72.3, SD 5.5 years) completed a modified Trail Making Test under 7 within-subject conditions: independent performance (baseline), 3 robot-assisted conditions, and 3 human-assisted conditions, each corresponding to low, medium, and high cognitive load levels. Performance accuracy and completion time were recorded as behavioral indicators. Perceived cognitive load was assessed using the National Aeronautics and Space Administration Task Load Index, and physiological stress was evaluated via pre- and postcondition salivary cortisol concentrations. Linear mixed-effects models were applied to examine main effects and interactions of age group, assistance type, cognitive load level, and time.

**Results:**

Significant interactions between age group and assistance type were observed for accuracy (*F*_1, 404.53_=6.50; *P*=.01) and perceived cognitive load (*F*_1, 403.45_=4.58; *P*=.03). Older adults demonstrated lower accuracy and higher perceived cognitive load during robot-assisted conditions compared with human-assisted conditions, whereas no such differences were observed in younger adults. Across age groups, human assistance improved performance at low and medium cognitive load levels. Physiological analysis revealed a significant age×assistance× time interaction (*F*_1, 156_=5.16; *P*=.02), with older adults showing increased posttask cortisol concentrations during robot-assisted interaction, indicating higher physiological stress.

**Conclusions:**

While both human and robotic assistance enhanced task performance relative to independent completion, the type of support critically shaped cognitive load responses in older adults. Robot-assisted interaction was associated with increased behavioral errors, higher perceived workload, and elevated physiological stress, suggesting that current SAR implementations may impose additional extraneous cognitive load in older users. These findings highlight the importance of designing adaptive, age-sensitive digital assistive systems that minimize cognitive burden through simplified interaction, responsive pacing, and multimodal support. Multimodal cognitive load assessment provides a valuable framework for optimizing the usability and effectiveness of assistive digital health technologies for aging populations.

## Introduction

Maintaining independence and cognitive efficiency in daily life constitutes a central objective in the promotion of active and healthy aging [1]. The ability to manage daily activities with adequate support has become a key focus across multiple disciplines, particularly in light of the projected rise in the global mean age over the coming decades [2]. In this context, health and social care systems are increasingly exploring digital health technologies to support autonomy, reduce care burden, and enable aging in place, underscoring the need for scalable and evidence-based interventions aimed at enhancing quality of life among older adults.

Technological progress has played a pivotal role in pursuing this goal. Intelligent systems can now be embedded within domestic environments, and embodied agents may be programmed to interact with users to accomplish shared objectives [3]. Within this framework, socially assistive robotics (SAR) leverages human-robot interaction (HRI) to facilitate such activities and to foster autonomy in everyday life [4]. SAR systems are increasingly positioned as interactive digital health interventions, integrating social interaction, guidance, and task support within real-world settings. However, recent evidence indicates that enabling communication with an artificial agent alone is insufficient to ensure effective interaction, particularly in vulnerable groups such as older adults [5]. Beyond demonstrating performance benefits, digital assistive technologies must also be evaluated in terms of usability, acceptability, and sustainability of long-term adoption [6,7]. Interventions that improve task execution but simultaneously increase cognitive or physiological strain may face significant barriers to real-world implementation.

From a cognitive ergonomics perspective, cognitive load (CL) represents a critical determinant of HRI effectiveness and of the usability and sustainability of digital health technologies [8]. Conceptually, CL is defined as the amount of mental resources required to perform a given task [9]. Because cognitive resources are inherently limited, individuals must distribute them efficiently across competing activities [10].

Importantly, CL is not a unitary construct but rather comprises three interrelated components: intrinsic, extraneous, and germane load. Intrinsic load stems from the inherent complexity of the task and the volume of information to be processed. Extraneous load arises from the presentation format and the presence of external distractions, both of which can be mitigated through effective task design. Germane load, instead, pertains to the cognitive processes that foster learning and schema construction. Recent theoretical models have proposed integrating intrinsic and germane load, conceptualizing intrinsic and extraneous load as the two principal dimensions of CL theory [11-13]. In applied digital health contexts, extraneous CL introduced by interface design is particularly problematic, as it may negate the intended supportive function of assistive technologies.

A growing body of research indicates that older adults face greater challenges in managing CL, thereby affecting their functional autonomy [14]. Such difficulties are often attributed to age-related cognitive decline, which manifests as increased fatigue and reduced efficiency in completing daily tasks [15]. These challenges extend to the use of technology, where the additional cognitive demands associated with complex interfaces further amplify CL [16,17]. Moreover, older adults’ attitudes toward advanced technologies are shaped not only by sociocultural factors but also by individual cognitive needs and the degree of system personalization [18]. Neglecting these factors’ risks imposing additional cognitive burdens and may contribute to digital exclusion among those most in need of support.

Despite the growing interest in SAR, few studies have directly compared robot-mediated assistance with human assistance under controlled CL conditions. Such comparisons are essential, as assistive technologies are often implicitly framed as substitutes for human support. Furthermore, assessing technology-assisted performance without an independent baseline condition limits the ability to determine whether observed effects reflect genuine support or task facilitation, as highlighted in previous work [19].

Accordingly, it becomes essential to determine whether technology use increases users’ CL, to identify the features responsible for such increases, and to examine how these effects differ across age groups. Such insights are crucial for the design of adaptive interfaces capable of dynamically accommodating users’ cognitive capacities. Nevertheless, the existing literature presents several methodological limitations in addressing these issues.

First, assessing CL during technology use without a baseline condition prevents reliable interpretation. Experimental designs should therefore include a comparison between technology-assisted and independent task performance to delineate the specific benefits and drawbacks of technological aids, a methodological framework exemplified by Varrasi et al [19]. Furthermore, studies asserting the positive impact of technology without incorporating a condition involving human support lack robustness; indeed, multiple aid conditions are required for a comprehensive evaluation. Likewise, hypothesizing user needs without conducting age comparisons constrains the development of genuine person-centered support systems. Specific sampling is thus necessary to enable between-group analyses and to characterize distinct population profiles. Finally, the reliance on a single CL measure limits construct validity, underscoring the need to use multimodal assessment approaches.

CL can be quantified through a range of behavioral, subjective, and physiological indicators, each providing complementary information on the cognitive demands placed on the individual. Behavioral indices include performance-based metrics, such as accuracy, error rates, and completion time, which serve as quantifiable markers of cognitive effort [20]. Subjective indices capture self-reported perceptions of mental workload, encompassing aspects such as perceived effort, frustration, and mental demand [21]. Integrating these approaches enables a comprehensive evaluation of cognitive demands and supports the robust assessment of assistive technologies in digital health contexts. Physiological indices, including heart rate variability, pupil dilation, blood and salivary cortisol concentrations, and electroencephalographic (EEG) activity, reflect the body’s physiological responses to cognitive strain and stress [22]. Integrating these methods offers a comprehensive assessment of CL and facilitates a more accurate evaluation of task design and learning efficiency.

Given these considerations, this study aimed to investigate whether, and in what ways, HRI and human-human interaction (HHI) influence CL management during task performance in younger and older adults. By incorporating an independent baseline condition and a multimodal psychometric framework, this study seeks to inform the design, evaluation, and deployment of age-sensitive digital assistive technologies.

## Methods

### Study Design

This study used a within-subject, experimental comparative design to examine differences in CL during independent, robot-assisted, and human-assisted task performance in younger and older adults.

### Participants

The study included 60 adult volunteers, comprising 30 younger adults (13 males, 17 females; mean age 34.8, SD 10.1 years) and 30 older adults (12 males, 18 females: mean age 72.3, SD 5.5 years), with sex distribution confirmed as balanced (*χ*²_1_=0.07; *P*=.79). Younger adults were aged 18‐45 years, and older adults were more than 65 years of age. In line with the study aims, age differed significantly between groups (*t*_58_=17.86; *P*<.001). Education levels were broadly comparable across groups. Among younger adults, 16.7% (5/30) reported high school education, 23.3% (7/30) bachelor’s, 30% (9/30) master’s, and 30% (9/30) PhD-level education. Among older adults, 33.3% (10/30) reported high school education, 30% (9/30) bachelor’s, 23.3% (7/30) master’s, and 13.3% (4/30) PhD-level education. Although younger adults showed a tendency toward higher educational attainment, this difference was not statistically significant (*χ*²_3_=4.09; *P*=.25). An a priori power analysis (2-tailed α=.05, 80% power) indicated that the sample size of 30 adults per group provides sufficient power to detect Cohen *d*∼0.74 for between-group comparisons and interaction effects (approximated as between-group differences in within-subject contrasts). Within-group paired contrasts have 80% power to detect dz∼0.53 (n=30) and dz∼0.37 when considering the full sample (N=60). These estimates represent conservative approximations relative to the mixed-effects modeling approach used in the primary analyses.

All participants underwent screening to confirm good general health, as only healthy individuals were included in the study. Specifically, participants had no prior diagnosis of neurological or psychiatric disorders and reported no previous direct experience interacting with socially assistive robots. This sampling strategy was adopted to isolate age-related differences in CL responses under controlled experimental conditions while minimizing additional variability associated with clinical impairment. Accordingly, the study was conceived as an initial step toward understanding how assistance modality influences CL, with the intention of extending this line of research to more vulnerable populations in future work.

### Ethical Considerations

The study was conducted in accordance with the ethical principles outlined in the Declaration of Helsinki. Ethical approval was obtained from the Nottingham Trent University Ethics Board (reference number 729) in March 2024. All participants provided written informed consent prior to participation and were informed of their right to withdraw at any time without consequence.

### Tools

The cognitive task used in this study was adapted from the B form of the Trail Making Test (TMT) [23]. The TMT was selected over alternative cognitive measures due to its engagement of multiple cognitive domains, including attention, visual search, executive functioning, and working memory. Consequently, the task required participants to simultaneously deploy multiple cognitive skills, closely reflecting the complexity of everyday activities.

**Figure 1.**
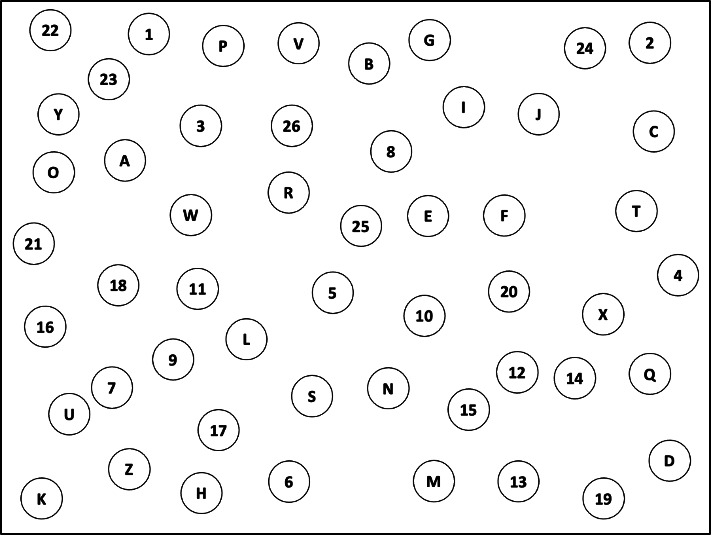
Cognitive task sheet used in the study. Example of one task version consisting of 52 randomly arranged circles distributed across a 29.7 × 42 cm sheet. Half of the circles contained English letters (A–Z), and the remaining circles contained numbers (1–26). Participants were instructed to connect the targets sequentially in alternating alphanumeric order (eg, 1–A–2–B–3–C) as quickly and accurately as possible. Seven task versions with different spatial arrangements were created to minimize learning effects across repeated conditions.

Specifically, the task used a 29.7×42 cm sheet of paper containing 52 randomly arranged circles (Figure 1). Half of the circles included English letters (A-Z), while the remaining circles contained numbers (1-26). A total of 7 task versions were created to minimize learning effects across repeated conditions. These versions were not intended to represent validated parallel forms of the TMT in a clinical sense; rather, they were task variants inspired by the TMT and designed to evoke comparable executive, attention, and visual search demands. For each version, the 52 circles were randomly distributed across the sheet to create distinct spatial arrangements while preserving the same basic task structure. Task versions were assigned randomly to participants across conditions, with each participant completing a different version in each condition. Objective performance was behaviorally evaluated based on the number of correctly connected pairs and the time required to complete the task (seconds).

The supportive agent used in this study was the NAO 6 humanoid robot (United Robotics Group). This platform was selected for its commercial availability and relevance to real-world assistive deployments aligned with the study’s requirements, including speech production, speech recognition, and social interaction. Previous studies have shown that NAO (Aldebaran, Paris, France) functions as an SAR that is generally well-received by older and vulnerable populations [16]. The robot is 58 cm tall and can interact through speech, gestures, and facial tracking. Programming was implemented using Choregraphe software (version 2.8.7; Aldebaran) together with Python (Python Software Foundation) to enable interactive collaboration with participants in 3 of the 7 experimental conditions.

The robot’s behavior was structured using block-based visual programming within a node flow interface. Interactions began with NAO tracking the participant’s face to simulate eye contact, followed by a self-introduction and delivery of initial task instructions. Speech recognition allowed the robot to repeat instructions upon participant request during an initial familiarization phase, which served as a vocal adaptation period responsive to participant feedback. After this phase, NAO did not repeat instructions to avoid disrupting task performance, although speech recognition remained active to manage pacing during the interaction. Indeed, the robot-participant interaction followed a structured and standardized dialogue logic designed to ensure consistency across participants while maintaining naturalistic turn-taking dynamics. Specifically, interaction sequences were organized into alternating robot-led instruction phases and participant execution phases. The robot presented task instructions verbally according to predefined scripts and advanced through task steps only after receiving explicit verbal confirmation from the participant (eg, “Okay” or “Done”), thereby regulating the interaction rhythm.

The presentation of task instructions varied systematically according to CL conditions. In the low-load condition, the robot delivered one pair of items at a time, whereas in the medium- and high-load conditions, multiple pairs were provided sequentially within a single instruction block. The interaction pacing was therefore partially scripted, with fixed instruction sequences, but temporally modulated by participant responses, allowing limited responsiveness to individual execution speed.

**Figure 2.**
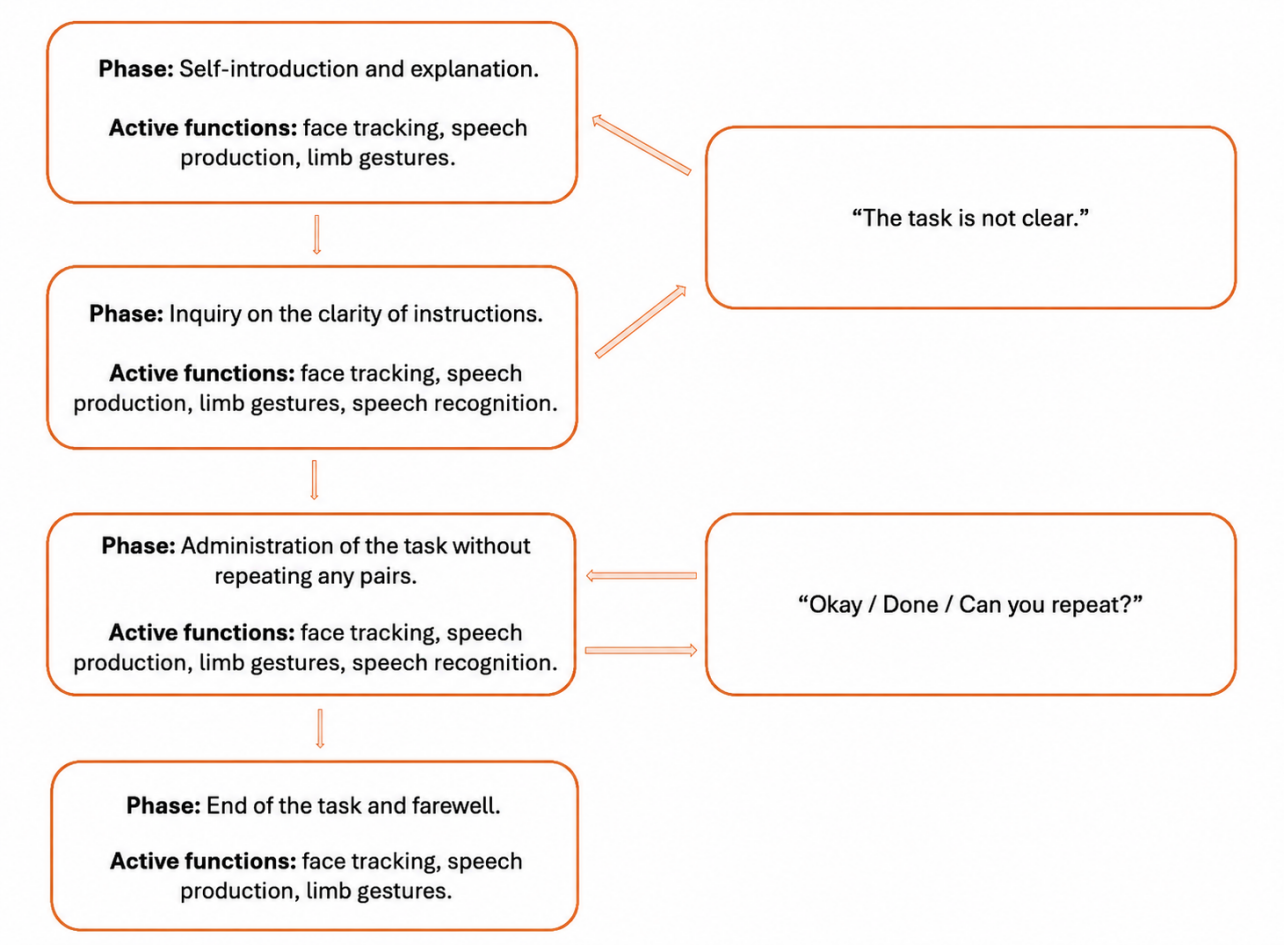
Flowchart of the interaction with the robot with active interfaces.

To ensure procedural reproducibility, the interaction logic combined fixed scripted content (instruction wording, sequence structure, and load manipulation) with controlled interaction triggers based on participant verbal feedback. A schematic representation of the interaction workflow is provided in Figure 2.

The flowchart illustrates the decision nodes governing instruction delivery, response confirmation, and transition between interaction phases, providing a visual representation of the standardized dialogue protocol implemented across conditions. Moreover, to enhance transparency and reproducibility of the interaction protocol, a structured overview of the robot dialogue architecture, including interaction phases, scripted instruction templates, and response-triggered transitions, is provided in Table 1.

**Table 1.** Structured overview of the robot dialogue architecture.

Phase	Robot action	Participant action	Trigger
Greeting	Introduces task	Listens	Experimenter
Instruction	Provides numbers and letters to connect	Executes task	Scripted
Confirmation	Waits for response	Verbal confirmation	Voice trigger
Transition	Provides next pair	Continues task	Confirmation

Perceived cognitive load (PCL) was assessed using the National Aeronautics and Space Administration Task Load Index (NASA-TLX) [24], a brief, self-administered psychometric tool evaluating 6 dimensions: mental demand, physical demand, temporal demand, performance, effort, and frustration. Each dimension was rated on a 20-point scale, with higher scores indicating greater perceived strain. The NASA-TLX was chosen for its robust psychometric properties and demonstrated superiority over alternative subjective workload measures [25], making it well-suited for assessing PCL in this study. In line with the primary aim of capturing overall perceived workload during assisted task performance, the total NASA-TLX score was used as a global indicator of subjective CL. Although the instrument allows examination of individual workload dimensions, the aggregated score has been widely used to provide a comprehensive estimate of perceived task demand, particularly in applied human-technology interaction research [25].

To objectively assess physiological stress responses associated with the CL tasks, saliva samples were collected immediately before and after each condition (ie, robot-assisted and human-assisted). Samples were obtained using a synthetic swab (Salivette Cortisol, Sarstedt), instructing participants to slowly move the swab around the oral cavity for 2 minutes, in accordance with the manufacturer’s instructions. All samples were maintained on crushed ice throughout testing, which did not exceed 45 minutes. Subsequently, saliva samples were centrifuged at 1000×*g* for 2 minutes (Espresso Microcentrifuge, Thermo Scientific), and the supernatant was transferred into 1.5 mL Eppendorf tubes and stored at −80 °C until analysis. Quantitative determination of salivary cortisol concentrations was performed using commercially available enzyme-linked immunosorbent assay kits (Salimetrics), following the manufacturer’s recommended procedures. Salivary cortisol was included as a physiological indicator of stress-related arousal associated with cognitive effort. While cortisol does not represent a direct measure of CL, it provides complementary information regarding the neuroendocrine response to task-related strain, allowing integration with behavioral and subjective workload indicators. Moreover, salivary cortisol was sampled only before and after each assisted block, and not after the independent baseline condition, because the physiological outcome was intended to capture the cumulative response associated with the two assistance modalities rather than transient changes across individual CL levels or during unassisted performance. This choice was based on both theoretical and methodological considerations, including the temporal dynamics of the hypothalamic-pituitary-adrenal axis, as salivary cortisol typically peaks approximately 20‐40 minutes after stress onset. Accordingly, cortisol was treated as a block-level marker of stress-related arousal, and restricting sampling to the assisted conditions helped reduce participant burden while preserving the feasibility and ecological validity of the experimental protocol.

### Procedure

Each participant completed the experimental protocol individually across seven conditions: one baseline condition performed independently, three robot-assisted conditions at varying CL levels (low, medium, and high), and three human-assisted conditions corresponding to the same CL levels.

The baseline condition was systematically administered at the beginning of the session to provide a common, unassisted reference point for all participants. Within each assisted block, CL levels were presented in a fixed progressive sequence (low, medium, and high) in both the robot-assisted and human-assisted conditions. This choice was made to ensure a gradual and controlled increase in task demands, thereby minimizing potential confusion associated with abrupt changes in difficulty and maintaining a stable and predictable interaction structure, particularly for older adults. In contrast, the order of the assisted blocks (robot-assisted vs human-assisted) was fully counterbalanced across participants to control for order effects at the level of assistance modality.

Both assisted conditions were administered within the same experimental session. Consequently, the precondition saliva sample for the second assisted block was collected immediately before its onset, following completion of the preceding condition and a brief transition phase required to reset the materials and prepare the subsequent task. While counterbalancing mitigates systematic sequence effects at the level of condition order, the absence of an extended washout interval implies that a residual carryover influence from the first assisted condition on the precondition cortisol value of the second block cannot be entirely excluded. The total duration of the experimental session was approximately 30 minutes.

After providing informed consent, the participant was seated at a desk and presented with the first task sheet. In the baseline condition, the participant was instructed to connect alternating numbers and letters in reverse order (ie, starting from the highest number and the last letter) as quickly and accurately as possible using a marker. This task was designed to elicit CL by engaging multiple cognitive domains, including semantic memory, working memory, visual search, and processing speed. Specifically, the participant was required to recall the reverse sequence of alphanumeric items, maintain this sequence in working memory, and identify and connect the corresponding circles on the sheet.

**Figure 3.**
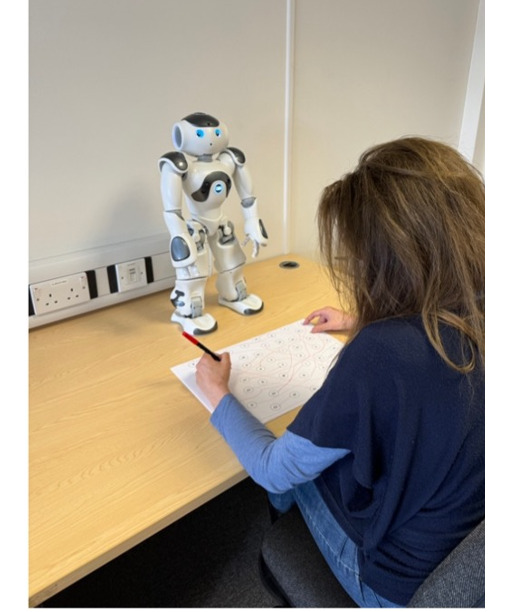
Setting of the robot-interactive task.

During the three robot-assisted conditions, the NAO humanoid robot acted as an interactive support agent (Figure 3). At the beginning of each assisted condition, the robot greeted the participant, introduced the task, and repeated the instructions upon request. Throughout task execution, the robot verbally provided the pairs of circles to be connected, thereby reducing reliance on semantic memory and allowing participants to focus on visual search and motor coordination. The robot advanced through the sequence based on participant responses, waiting for a verbal confirmation (eg, “Okay”) before announcing the next pair. To prevent learning effects, the sequence of pairs was randomized across trials. CL was systematically manipulated as follows: in the low-load condition (second and fifth condition), the robot presented one pair at a time; in the medium-load condition (third and sixth condition), two to four pairs were provided simultaneously; and in the high-load condition (fourth and seventh condition), five to seven pairs were delivered concurrently. The robot did not repeat any pair throughout the session.

The human-assisted condition was administered by a trained experimenter who followed a standardized script designed to mirror the robot-assisted protocol in content, sequence, and CL manipulation. The experimenter delivered the instructions verbally using the same wording as the robot prompts, maintained the same load-specific structure, and waited for the same participant verbal confirmations before proceeding to the next step. A brief initial familiarization phase was also included to ensure procedural parity with the robot-assisted condition: during this phase, the experimenter presented the task instructions and allowed participants to request clarification, analogous to the vocal adaptation period implemented in the HRI condition. Following familiarization, the interaction proceeded according to the predefined script without additional instruction repetition. The only difference between conditions was the modality of delivery, with all other procedural elements held constant. A structured overview of the human-assisted dialogue, emphasizing the fidelity rules for the adherence to the HRI condition, is provided in Table 2.

**Table 2.** Structured overview of the human dialogue architecture with fidelity rules.

Phase	Human experimenter action	Participant action	Trigger and fidelity rule
Greeting	Introduces task	Listens	Standardized opening script
Instruction	Provides numbers and letters to connect	Executes task	Verbatim script
Confirmation	Waits for response	Verbal confirmation	Proceed only after confirmation
Transition	Provides next pair	Continues task	Same sequencing as robot condition

At the end of each condition, including baseline, robot-assisted, and human-assisted tasks, participants completed the NASA-TLX questionnaire to evaluate PCL. As described previously, saliva samples were collected at the beginning and the end of both the robot-assisted and human-assisted conditions to capture physiological markers of CL, yielding four samples per participant.

For data analysis, two independent experimenters (SV and RV) assessed task performance across all conditions. Performance measures included accuracy (number of correctly connected pairs) and completion time (in seconds). Subjective workload was quantified using NASA-TLX scores, while physiological responses were evaluated by comparing pre- and postcondition cortisol concentrations across the robot- and human-assisted settings.

### Statistical Analyses

Linear mixed-effects models were applied using restricted maximum likelihood estimation to examine differences across the primary outcome variables, including NASA-TLX scores, task performance indices, and cortisol responses. Participants were included as random intercepts to account for interindividual variability in baseline performance and physiological measures.

A type III ANOVA with Satterthwaite approximation was used to assess the main effects and interactions of condition (robot-assisted vs human-assisted), CL level (baseline, low, medium, and high), and age group (younger vs older adults). Condition at varying load levels was modeled as a within-subject factor, while age group was treated as a between-subject factor.

Post hoc comparisons were performed on estimated marginal means and mean differences (M_dif_) to further explore significant main effects and interactions. Tukey-adjusted estimated marginal means comparisons were used to correct for multiple testing. In line with the study’s objectives, particular attention was devoted to the interaction between age group and experimental condition to clarify differential patterns of CL management and task performance across age cohorts.

## Results

Mean (SD) values for accuracy (number of correctly matched pairs), completion time (in seconds), and PCL across the seven experimental conditions, as well as cortisol levels (nmol/L) measured before and after the robot-assisted and human-assisted conditions, are presented in Tables3  4.

**Table 3.** Task performance and perceived cognitive load.

Conditions	Accuracy, mean (SD)	Time, mean (SD)	PCL^a^, mean (SD)
Alone			
Baseline	23.4 (5.8)	405 (116)	49.2 (15.1)
Robot			
Low CL^b^	24.4 (2.5)	364 (141)	40.9 (16.6)
Medium CL	16 (4)	342 (69.6)	58.3 (15.2)
High CL	9.0 (3)	251 (56)	66.2 (14)
Human			
Low CL	25.7 (0.6)	332 (91.4)	40.3 (15.3)
Medium CL	18.4 (3.2)	362 (86.5)	57.1 (13.4)
High CL	8.2 (2.3)	246 (61.1)	68.5 (12.3)

a PCL: perceived cognitive load.

b CL: cognitive load.

**Table 4.** Cortisol levels^a^ by condition and time.

Conditions and time		Cortisol, mean (SD)
Human		
Pre		7.72 (8.54)
Post		7.48 (8.09)
Robot		
Pre		6.02 (3.51)
Post		8.87 (10.4)

a Cortisol levels (nmol/L) were measured at the beginning (pre) and end (post) of the human-human interaction and human-robot interaction conditions.

**Figure 4.**
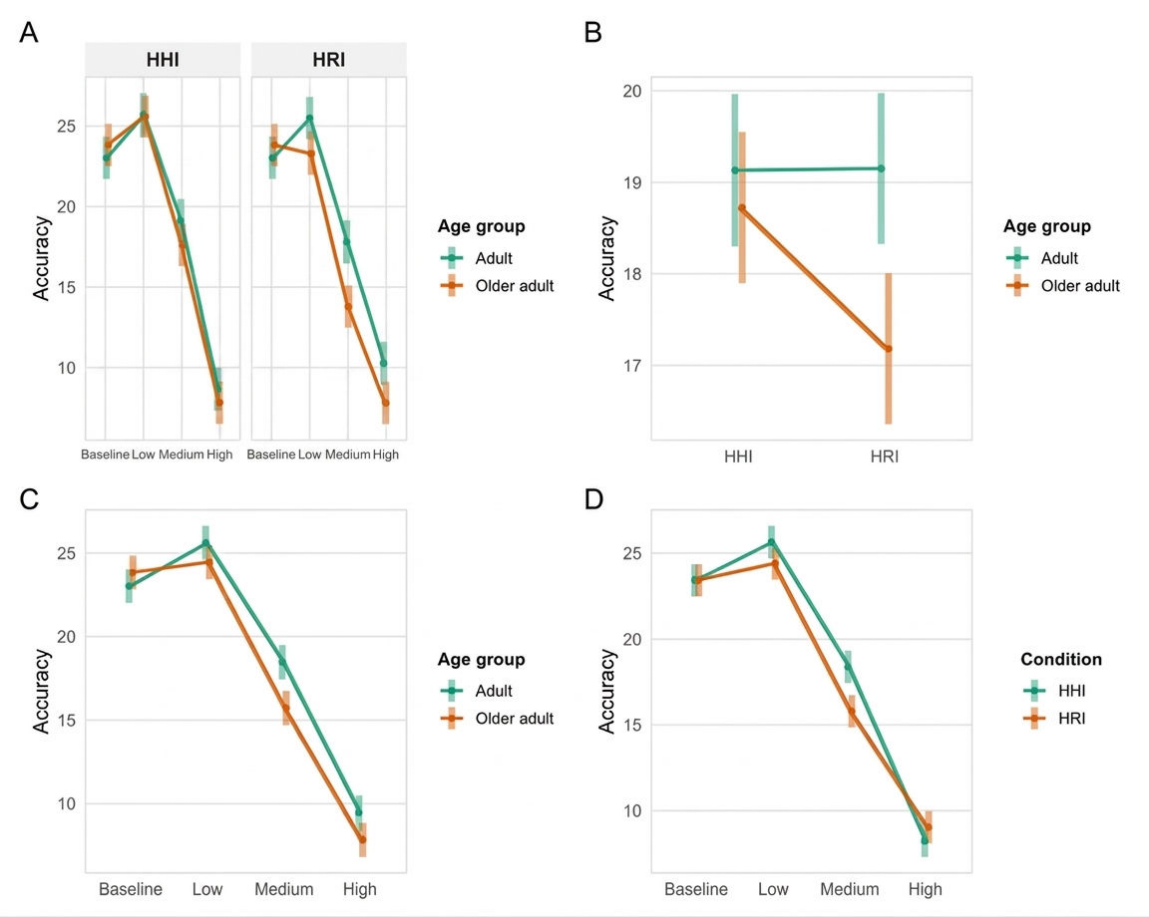
Older adults had a worse performance at HRI condition than younger adults (**A**) and than HHI (**B**). Lower accuracy was shown at increasing levels of cognitive load with respect to adults (**C**), while high cognitive load was challenging for all participants independently from the interactive condition (**D**). HHI: human-human interaction; HRI: human-robot interaction.

Regarding performance, analysis of accuracy revealed significant interactions between age group and condition (*F*_1, 404.53_=6.50; *P*=.01) as well as between age group and CL level (*F*_3, 403.45_=5.88; *P*<.001). Post hoc comparisons indicated that older adults exhibited lower accuracy in the HRI condition compared with younger adults (M_dif_=1.97, SE=0.58; *t*_106_=3.34; *P*=.001; Figure 4A) and also relative to the human-assisted condition (M_dif_=1.54, SE=0.43; *t*_403_=3.57; *P*<.001; Figure 4B). Additionally, older adults demonstrated significantly lower accuracy than younger adults at medium (M_dif_=2.75, SE=0.73; *t*_219_=3.75; *P*<.001) and high CL levels (M_dif_=1.64, SE=0.73; *t*_219_=2.23; *P*=.03), whereas no significant differences were observed at low load (M_dif_=1.16, SE=0.73; *t*_219_=1.58; *P*=.12) or in the baseline condition (M_dif_=−0.80, SE=0.73; *t*_217_=−1.09; *P*=.27; Figure 4C).

A significant interaction between condition and CL level was also observed (*F*_1, 403.45_=5.83*; P*<.001). Across all participants, post hoc tests revealed higher accuracy in the human-assisted compared with the robot-assisted condition at low (M_dif_=1.25, SE=0.61; *t*_404_=2.05; *P*=.04) and medium CL levels (M_dif_=2.58, SE=0.61; *t*_404_=4.21; *P*<.001), whereas no significant differences emerged at high CL (M_dif_=−0.79, SE=0.61; *t*_404_=−1.29; *P*=.19; Figure 4D).

**Figure 5.**
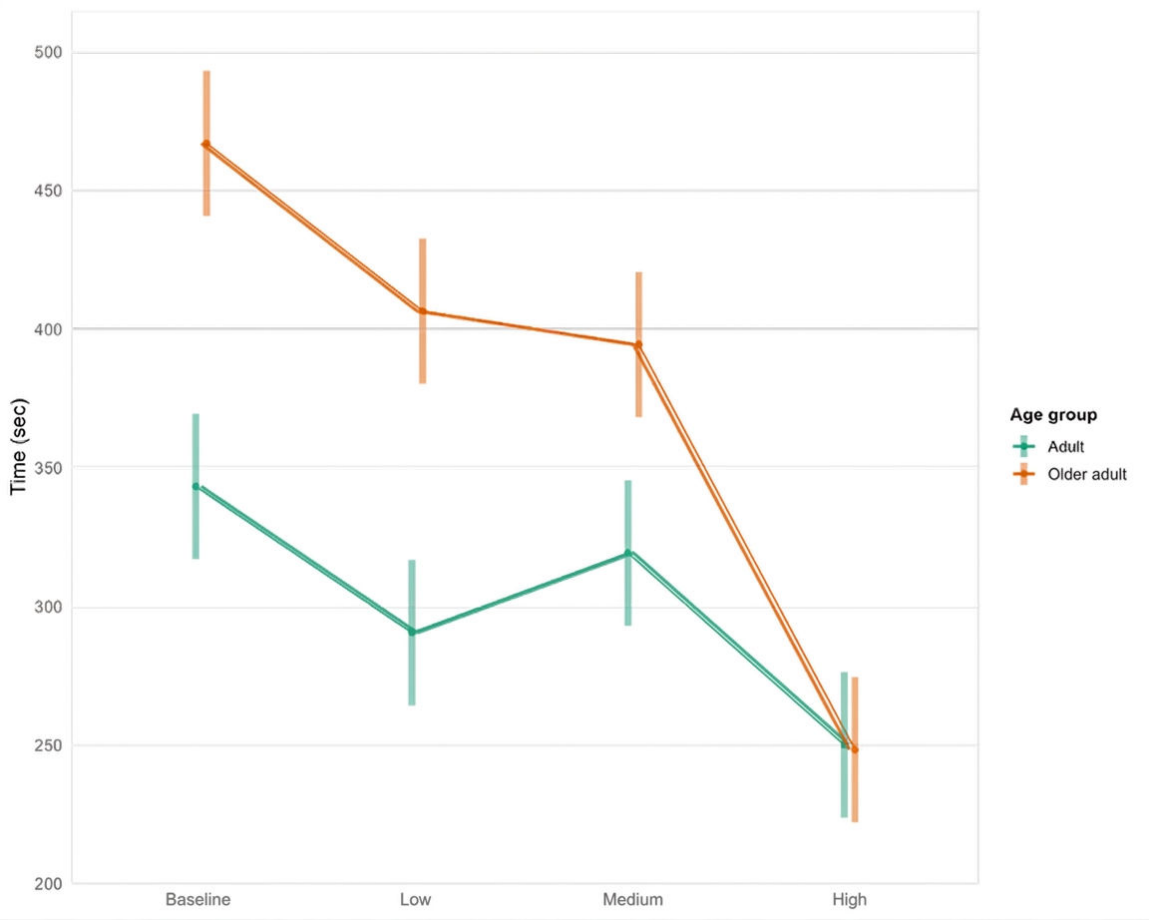
Younger adults were faster than older adults at the baseline, low cognitive load, and medium cognitive load.

Analysis of task completion time revealed a significant interaction between age group and CL level (*F*_3, 402.85_=23.73; *P*<.001). Post hoc comparisons indicated that younger adults completed the task faster than older adults at baseline (M_dif_=−123.73, SE=18.70; *t*_114_=−6.62; *P*<.001), as well as under low (M_dif_=−115.95, SE=18.70; *t*_115_=−6.19; *P*<.001) and medium CL conditions (M_dif_=−75.16, SE=18.70; *t*_115_=−4.01; *P*<.001). No significant difference was observed at high CL (M_dif_=1.73, SE=18.70; *t*_115_=0.09; *P*=.92; Figure 5).

**Figure 6.**
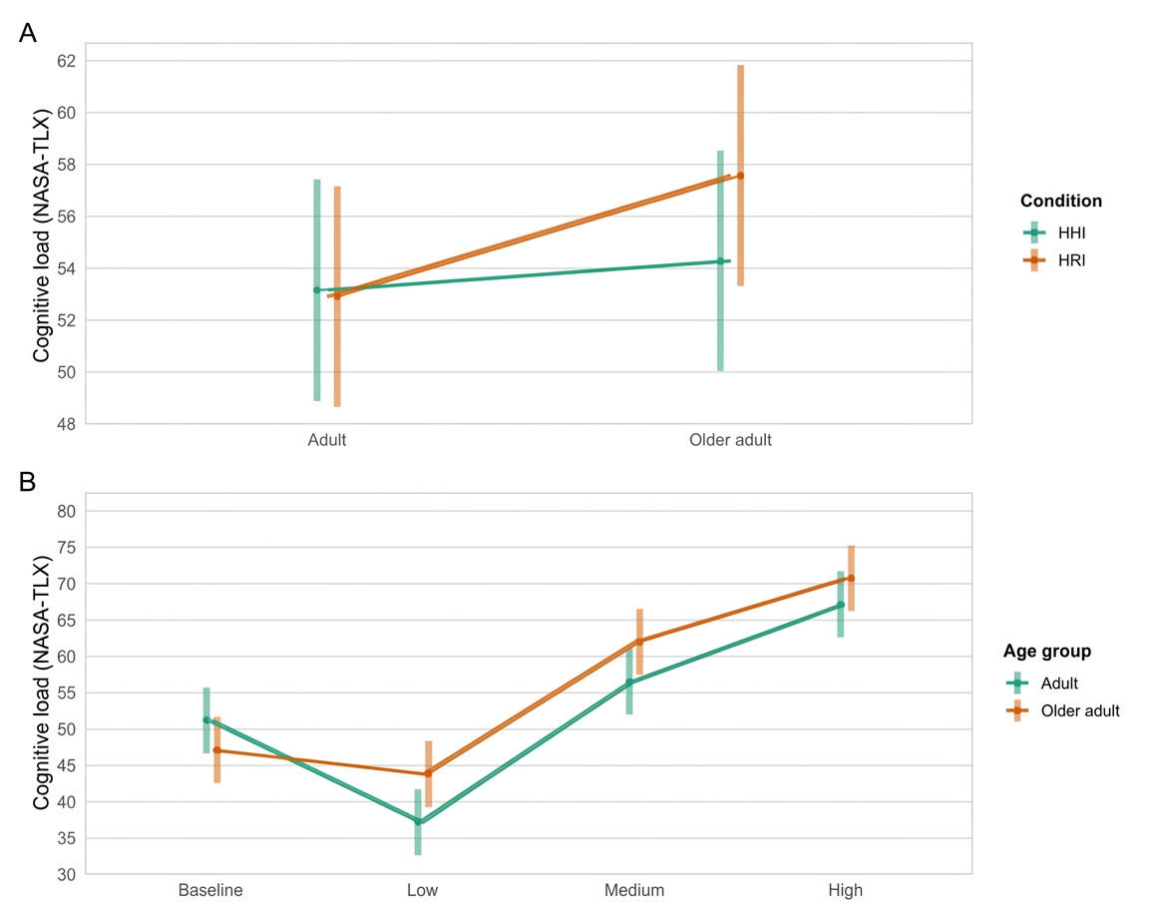
Older adults reported significantly higher overall cognitive load during the robot condition compared to the human condition. No significant condition differences were found for younger adults (**A**). Older adults perceived greater cognitive load than adults specifically under low cognitive load levels (**B**). HHI: human-human interaction; HRI: human-robot interaction; NASA-TLX: National Aeronautics and Space Administration Task Load Index.

Analysis of NASA-TLX scores revealed significant interactions between age group and condition (*F*_1, 403.45_=4.58; *P*=.03) as well as between age group and CL level (*F*_3, 403.12_=8.53; *P*<.001). Regarding the interaction between age group and condition, post hoc comparisons showed that older adults reported significantly higher scores during the robot-assisted condition (mean 57.60, SE 2.13) compared with the human-assisted condition (mean 54.3, SE 2.13), with an estimated M_dif_ of −3.29 (SE=1.16; *t*_403_=−2.83; *P*=.005). No significant difference was observed in younger adults (M_dif_=0.25, SE=1.18; *t*_404_=0.21; *P*=.83), indicating that older adults experienced greater perceived CL when interacting with the robot compared with human support (Figure 6A).

For the interaction between age group and CL level, post hoc analysis revealed that older adults reported significantly higher total scores at the low CL level (mean 43.8, SE 2.28) compared with younger adults (mean 37.2, SE 2.29; M_dif_=−6.63, SE=3.23; *t*_89.0_=−2.05; *P*=.04), suggesting that older adults perceive more CL than younger adults under low-load conditions (Figure 6B).

**Figure 7.**
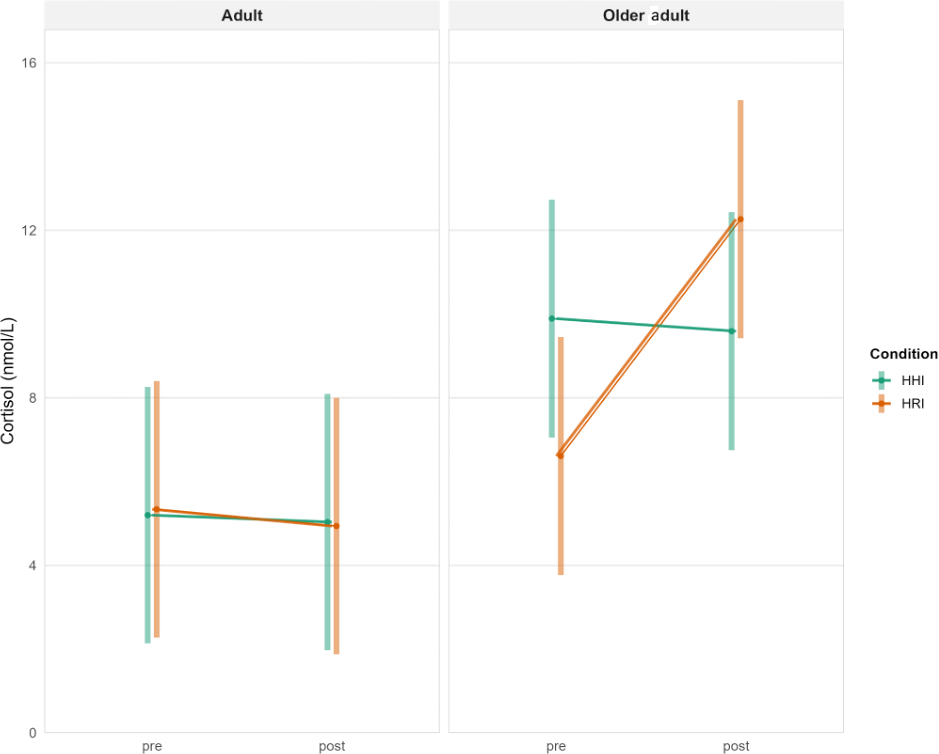
Salivary cortisol concentrations (nmol/L) at pre- and post-HHI and HRI, divided by age groups. Older adults showed an increase of cortisol after the robot condition. HHI: human-human interaction; HRI: human-robot interaction.

Analysis of the physiological measure of salivary cortisol concentration revealed a significant interaction between age group, condition, and time (pre vs post*; F*_1, 156_=5.16; *P*=.02). When analyzed separately by age group, older adults demonstrated a significant interaction between condition and time (*F*_1, 156_=10.32; *P*=.001), whereas younger adults showed no significant effect (*F*_1, 156_=0.01; *P*=.90). Post hoc comparisons indicated that older adults exhibited a significant increase in cortisol concentrations from baseline to postintervention during the HRI condition (M_dif_=−5.65, SE=1.31; *t*_156_=−4.31; *P*=.001; Figure 7).

## Discussion

### Principal Findings

This study aimed to investigate how HHI and HRI support the management of CL during task performance in younger and older adults, integrating behavioral, subjective, and physiological measures. By including a baseline condition without assistance and systematically varying CL levels, this research extends the literature on cognitive ergonomics in aging and technology-assisted contexts by providing an applied evaluation of assistive support modalities that are increasingly proposed as digital health solutions for aging populations. Importantly, these findings should also be interpreted within a translational digital health perspective. While assistive technologies are frequently evaluated in controlled experimental settings, their successful implementation depends on sustained usability, user acceptance, and ecological integration into daily routines. In this context, increases in CL or physiological load, even when accompanied by performance improvements, may represent critical barriers to long-term engagement and adherence. Consequently, evaluating assistive systems through a multidimensional framework that captures both performance outcomes and user burden is essential to inform scalable and sustainable deployment strategies.

### Behavioral Measures

Consistent with previous evidence indicating age-related declines in processing speed and working memory capacity [26,27], older adults demonstrated lower accuracy and required more time to complete tasks as CL increased. These differences were most pronounced at medium- and high-load levels, supporting theoretical frameworks positing that increased task complexity disproportionately affects older adults due to age-related cognitive changes [28]. Notably, at low CL levels, accuracy differences between age groups were reduced, suggesting that minimal task demands may better align with preserved cognitive capacities in older adults, enabling performance comparable to that of younger adults. Importantly, these findings reinforce the need to evaluate assistive technologies for older adults not only under simplified or optimal conditions, but also across varying levels of cognitive demand that more closely reflect real-world task complexity.

The interaction between type of assistance and CL revealed that HHI generally supported higher accuracy compared with HRI, particularly at low- and medium-load levels. This finding aligns with research suggesting that human support can more flexibly and adaptively accommodate the nuanced needs of older adults, thereby reducing extraneous CL [29]. The absence of significant accuracy differences between HRI and HHI at high CL may tentatively reflect a ceiling effect, whereby performance approached an upper limit, reducing sensitivity to differences between assistance modalities. However, this interpretation should be treated with caution, as performance saturation was not formally tested in this study.

### Subjective Measures

Subjective measures of PCL, assessed via NASA-TLX scores, indicated that older adults consistently reported higher CL during HRI compared with HHI, particularly under low-load conditions. This finding is especially relevant from a usability and adoption perspective, as increased perceived workload under otherwise manageable task demands may negatively influence trust, acceptance, and sustained engagement with assistive technologies. This result suggests that even minimal interactions with robotic agents can impose additional extraneous cognitive demands for older adults, potentially due to challenges in speech recognition, turn-taking, or the social-cognitive demands of interpreting robotic behavior [30]. It is important to acknowledge that certain technical constraints are inherent to current HRI systems. Features such as response latency, variability in speech recognition, and predefined interaction pacing represent realistic operational characteristics of SARs rather than limitations of the experimental design. These factors may require users to allocate additional attentional and executive resources to manage turn-taking and system feedback, thereby contributing to increased extraneous CL. Such demands may be particularly relevant for older adults, who may be more sensitive to temporal unpredictability and reduced interactional flexibility. Recognizing these constraints as ecological features of present technologies highlights key targets for future system refinement, including more robust speech processing and adaptive interaction pacing, which may ultimately improve usability and reduce cognitive burden. By contrast, younger adults showed no differences in PCL between assistance types, suggesting that HRI may be more readily accommodated by individuals with higher baseline cognitive flexibility and greater familiarity with technology [31].

### Physiological Measures

Physiological measures of salivary cortisol concentrations corroborated these findings, revealing increased cognitive strain during HRI for older adults, with significant postintervention elevations observed in the HRI condition. This is consistent with prior literature demonstrating heightened physiological reactivity in older adults to tasks perceived as challenging or unfamiliar [32]. The convergence of behavioral, subjective, and physiological indicators strengthens the interpretation that HRI, in its current form, may elicit latent stress responses in older users that are not fully captured by performance metrics alone, underscoring the value of multimodal assessment frameworks in digital health evaluation. Indeed, the use of the total NASA-TLX score allowed the capture of participants’ global perception of workload across interaction modalities, providing an ecologically valid estimate of overall task burden. Complementarily, cortisol responses were interpreted as markers of physiological stress associated with cognitive effort rather than direct indicators of CL per se. The consistency of these measures supports a multidimensional interpretation of user strain during assistive interaction. The absence of similar effects in younger adults underscores the differential impact of HRI on cognitive stress across age groups and highlights the importance of integrating physiological, subjective, and performance-based measures to comprehensively assess CL.

### Future Perspectives and Limitations

Together, these findings highlight the complex interplay among aging, CL management, and the type of assistance provided during task performance. Previous research on behavioral and cognitive-affective regulation has similarly emphasized the need for individualized and context-sensitive approaches in psychological assessment and intervention [33,34]. Within the context of assistive digital technologies, these results suggest that “one-size-fits-all” interaction models are unlikely to meet the needs of older adults with heterogeneous cognitive profiles. While robotic assistance offers scalable and consistent support, its current implementation may inadvertently increase extraneous CL for older adults, particularly in tasks requiring social-cognitive engagement. In contrast, human assistance appears to mitigate these challenges, supporting better performance and lower perceived strain under comparable task demands.

From a digital health design and deployment perspective, these findings suggest that SAR systems should prioritize the minimization of extraneous CL through simplified interaction structures, improved speech recognition robustness, and adaptive pacing strategies that respond to individual user performance and stress indicators. Incorporating real-time or near–real-time indicators of CL could enable dynamic adjustment of assistance, reducing unnecessary cognitive strain and improving user experience. Moreover, hybrid models that combine robotic assistance with intermittent human support may represent a pragmatic transitional approach, particularly in early stages of adoption or in cognitively demanding tasks. These considerations are particularly relevant for large-scale implementation strategies, where technologies must remain cognitively sustainable across prolonged and repeated use in real-world environments.

Beyond implications for system design and deployment, this study also offers methodological insights that strengthen the interpretation of technology-assisted CL effects. Importantly, the inclusion of an independent baseline condition in this study strengthens the interpretation of the observed effects by enabling direct comparison between assisted and unassisted task performance. This design feature allows differentiation between genuine support-related benefits and task facilitation effects that may arise simply from external guidance, irrespective of the modality of assistance. By contextualizing both human and robotic assistance against independent performance, the findings demonstrate that while assistance generally improves task outcomes, the cognitive and experiential costs associated with different support modalities vary substantially, particularly for older adults. An additional methodological strength is that the human-assisted condition was operationalized through a standardized script matched to the robot-assisted protocol in wording, sequencing, and load manipulation. This design choice reduced the risk that differences between conditions were attributable to variability in human delivery style rather than to the assistance modality itself. This comparative framework enhances internal validity and provides a more robust basis for evaluating assistive technologies as potential substitutes or complements to human support in real-world digital health contexts.

The observed interaction patterns suggest that technological interventions designed for older adults should prioritize reducing extraneous CL by simplifying robot interfaces, improving speech recognition accuracy, and implementing adaptive pacing tailored to individual users. Indeed, although older adults view social robots as a potential tool to support their daily activities, our results further highlight the importance of designing such systems according to users’ specific needs [5]. Additionally, multimodal support strategies, including visual cues and simplified verbal prompts, may enhance the usability and effectiveness of HRI by aligning it more closely with the cognitive profiles of older adults.

This study suffers from some limitations. For instance, the generalizability of these findings. Although the inclusion of healthy younger and older adults allowed us to examine age-related differences under controlled conditions, the results cannot be directly generalized to more vulnerable groups, such as individuals with mild cognitive impairment or other clinical conditions for whom SARs may be particularly relevant. Future studies should therefore replicate this design in clinical and functionally vulnerable populations to determine whether the observed patterns of behavioral, subjective, and physiological response are preserved, attenuated, or amplified in contexts of greater cognitive vulnerability. Then, the seven task versions were not formally tested for equivalence in difficulty. Accordingly, they should not be interpreted as psychometrically validated parallel forms of the TMT. Although random assignment of versions across participants and conditions was used to reduce systematic version effects and support internal validity, residual differences in difficulty between task versions cannot be entirely excluded. Future studies should include formal equivalence testing of task variants or adopt a fully validated parallel-form procedure if the task is to be used repeatedly across conditions. A further methodological consideration concerns the fixed progression of CL levels within each assisted block. Although this approach ensured a controlled and gradual increase in task demands and supported procedural consistency across participants, it does not allow complete separation of CL effects from potential sequence effects. Future studies may benefit from counterbalancing or randomizing load sequences, although such designs should carefully consider the potential impact of increased task-switching demands, particularly in older populations. An additional limitation concerns the interpretation of the nonsignificant differences in accuracy between conditions at high CL. Although a ceiling effect may provide a plausible explanation, this possibility was not directly tested through specific analyses of performance saturation. As such, this interpretation should be considered tentative, and future studies should include measures or analytical approaches specifically designed to detect ceiling effects. Moreover, the sequential structure of the assisted blocks should be considered when interpreting the cortisol findings. The order of robot-assisted and human-assisted conditions was fully counterbalanced, which reduced the likelihood that the results were driven by presentation order. However, because the precondition cortisol sample for the second block was collected immediately after completion of the first assisted condition, a residual carryover effect cannot be completely excluded. This limitation is particularly relevant for salivary cortisol, given its temporal dynamics and slower recovery profile. Future studies may benefit from longer recovery intervals or separate-session administration to further reduce potential carryover effects while preserving the advantages of counterbalanced designs. Another important methodological consideration is that salivary cortisol was assessed only in the assisted conditions and at the block level, whereas behavioral and subjective outcomes were collected across all load levels, including baseline. Consequently, the physiological findings should be interpreted as reflecting differences between robot-assisted and human-assisted interaction, rather than as load-specific effects or as changes relative to the independent baseline condition. This design choice was consistent with the intended use of cortisol as a cumulative marker of stress-related arousal; however, it also limits direct comparison between physiological and other outcome domains and should be considered when interpreting the multimodal findings.

Future research should examine longitudinal exposure to robotic systems to determine whether increased familiarity reduces cognitive and physiological strain in older adults, potentially enhancing acceptance and efficacy. Such work is essential to distinguish short-term novelty or learning effects from stable interaction patterns that are likely to emerge during real-world deployment. Expanding physiological monitoring to include measures such as heart rate variability and electroencephalography could further elucidate the real-time cognitive dynamics associated with HRI and HHI across age groups.

### Conclusions

In conclusion, this study provides important evidence regarding CL management in aging populations within assisted task contexts, emphasizing the need to tailor support strategies to individual cognitive capacities. While HRI holds promise for facilitating aging-in-place initiatives, its effectiveness depends on careful interaction design and adaptive support mechanisms that ensure robotic assistance reduces, rather than amplifies, cognitive and physiological burden. Optimizing these systems is critical to supporting autonomy, usability, and quality of life in the context of active aging. Although both HHI and HRI improved performance compared with independent task completion, the effectiveness of assistance was strongly dependent on the type and complexity of support, particularly in older adults. Human assistance consistently supported higher accuracy and lower perceived workload, whereas robotic assistance, despite its promise for scalable and consistent support, was associated with increased perceived and physiological CL in older adults, especially under low and medium task demands. Overall, these findings highlight the importance of designing adaptive, age-sensitive digital assistive systems that minimize cognitive burden through simplified interaction, responsive pacing, and multimodal support. Importantly, even when assistive technologies improve task performance, increases in CL or physiological load may limit usability, scalability, and sustainable real-world deployment in aging populations. By demonstrating the value of multimodal CL assessment and tailored support strategies, this study provides actionable evidence to guide the design, evaluation, and deployment of assistive technologies that genuinely promote autonomy and quality of life in the context of active aging.

## Abbreviations

CL: cognitive load
HHI: human-human interaction
HRI: human-robot interaction
M_dif_: mean difference
NASA-TLX: National Aeronautics and Space Administration Task Load Index
PCL: perceived cognitive load
SAR: socially assistive robotics
TMT: Trail Making Test
